# Chemical Synthesis, Characterization, and Biocompatibility Study of Hydroxyapatite/Chitosan Phosphate Nanocomposite for Bone Tissue Engineering Applications

**DOI:** 10.1155/2009/512417

**Published:** 2009-01-25

**Authors:** Nabakumar Pramanik, Debasish Mishra, Indranil Banerjee, Tapas Kumar Maiti, Parag Bhargava, Panchanan Pramanik

**Affiliations:** ^1^Department of Chemistry, Indian Institute of Technology Kharagpur, Kharagpur 721302, India; ^2^Department of Biotechnology, Indian Institute of Technology Kharagpur, Kharagpur 721302, India; ^3^Metallurgical Engineering and Materials Science, Indian Institute of Technology Bombay, Mumbai 400076, India

## Abstract

A novel bioanalogue hydroxyapatite (HAp)/chitosan phosphate (CSP) nanocomposite has been synthesized by a solution-based chemical methodology with varying HAp contents from 10 to 60% (w/w). The interfacial bonding interaction between HAp and CSP has been investigated through Fourier transform infrared absorption spectra (FTIR) and x-ray diffraction (XRD). The surface morphology of the composite and the homogeneous dispersion of nanoparticles in the polymer matrix have been investigated through scanning electron microscopy (SEM) and transmission electron microscopy (TEM), respectively. The mechanical properties of the composite are found to be improved significantly with increase in nanoparticle contents. Cytotoxicity test using murine L929 fibroblast confirms that the nanocomposite is cytocompatible. Primary murine osteoblast cell culture study proves that the nanocomposite is osteocompatible and highly in vitro osteogenic. The use of CSP promotes the homogeneous distribution of particles in the polymer matrix through its pendant phosphate groups along with particle-polymer interfacial interactions. The prepared HAp/CSP nanocomposite with uniform microstructure may be used in bone tissue engineering applications.

## 1. Introduction

Hydroxyapatite (Ca_10_(PO_4_)_6_(OH)_2_) (HAp) is an interesting biomaterial with potential orthopedic, dental, and maxillofacial applications due to its excellent biocompatibility, bioactivity, and osteoconductivity [1]. HAp, being chemically and structurally similar to the inorganic component of bone, enamel, and dentin has received considerable attention from the biologists and biomaterial scientists. It has been successfully used as bone fillers, aesthetic restorative, coating of orthopedic implants, filler of inorganic/polymer composites, cell-culture carriers, and so on. It is, however, worth-mentioning that the application of pure HAp is being limited, due to its brittleness. In recent years, the development of bioactive ceramic-polymer composites commonly known as bioanalogue has gained a phenomenal impetus in the orthopedic field for their bone analogue design as well as good biological and mechanical performances to meet specific clinical requirements [2–4]. The idea is to use a ceramic-polymer composite material that can develop a considerable anisotropic character by means of adequate orientation techniques reinforced with a ceramic that simultaneously assures the mechanical reinforcement and the bioactive character of the implant [4–7]. In fact, natural bone tissue is a biocomposite, composed of nano-hydroxyapatite (n-HAp) crystals dispersed in a collagen matrix.

In the design of hydroxyapatite-based bioanalogue composites, the most commonly used matrices include polymers like poly(methyl methacrylate) (PMMA), high-density polyethylene (HDPE), poly-L-lactide (PLA), and many other. However, the natural biopolymers have received much attention in the fields of orthopedic and other biomedical applications, due to their excellent biocompatibility and biodegradability [8]. Chitosan (poly-1,4-D-glucosamine), a partially deacetylated form of chitin, is structurally similar to glycosaminoglycan, and has many desirable properties as tissue engineering scaffolds [9].

The mechanical properties of an HAp/polymer composite can be significantly improved by controlling the interfacial bonding between matrix and the reinforcement; and without proper interface control, a brittle, polyphase material results rather than a toughened composite. Various methodologies have been developed to improve the interfacial bonding between the same [10, 11]. Nevertheless, the use of judiciously chosen coupling/anchoring agents has been proved as a reliable method to strengthen the interfacial bonding between the filler and polymer matrix with an appreciable enhancement of the compatibility by making the chemical bridges between the same. Organosilane- and organotitanate-based coupling agents have already been reported to tailor the particle surface properties by lowering their specific surface energy [12, 13]. Recently, grafting of organophosphorus coupling agents (OPCAs) on preformed inorganic supports or in-situ formation of the inorganic part in presence of organophosphorus reagents offers a potential alternative to the silicon- or titanate-based coupling agents. Based on the ability of phosphonate ions to exchange with the phosphate ions on HAp crystals, any polymer containing a number of phosphate or phosphonic acid groups is expected to have higher affinity for the HAp particles [14]. Moreover, the phosphate-containing polymers are expected to have higher mechanical properties and biocompatibility [15]. In the preparation of bioanalogue composites, organophosphonate- or phosphate-based coupling/anchoring agents are being extensively used by the researchers to improve the compatibility between inorganic nanoparticles and polymer matrix [16–20]. Greish and Brown have developed a biocompatible HAp-Ca poly(vinyl phosphonate) composite for clinical applications [14]. Varma et al. have reported the preparation of calcium phosphate-coated phosphorylated chitosan film by a biomimetic method [18]. Tanaka et al. have reported the synthesis of surface-modified calcium hydroxyapatite with pyrophosphoric acid for use as bioceramics, particularly for orthopedic applications [19]. Choi et al. have reported the preparation of surface-modified hydroxyapatite nanocrystals by grafting of polymers containing phosphonic acid groups [20]. Phillips et al. have grafted allyl phosphonic acid on calcium phosphate to produce a chemically bonded composite with superior mechanical properties [21]. All these studies unequivocally suggest that phosphonate/phosphoric acid-based coupling agents can be successfully employed to enhance the interfacial bonding between the particles and the polymer matrix, and hence to improve the mechanical properties of the resulting composites.

In the present paper, we have attempted to prepare hydroxyapatite (HAp)/chitosan phosphate (CSP) nanocomposite through a simple solution-based chemical method, which has the potential of providing much better dispersion of n-HAp particles in the polymer matrix yielding a composite with uniform microstructure. CSP has been chosen as polymer matrix because phosphate groups of CSP can act as coupling/anchoring agent, which is a good cation absorber and has higher affinity towards the n-HAp particles [15]. Moreover, it is easy to prepare from cheaper ingredients. We have also extensively studied the dispersion, morphology, nanoparticle-polymer matrix bonding interactions, mechanical properties, as well as in vitro biocompatibility and osteogenicity of the synthesized HAp/CSP nanocomposite.

## 2. Materials and Methods

### 2.1. Chemicals

Calcium nitrate (Ca(NO_3_)_2_ · 4H_2_O) (99%), diammonium hydrogen phosphate ((NH_4_)_2_HPO_4_) (DAHP) (99%), ammonia solution (25%), ammonium chloride (98%), triethanolamine (TEA), and orthophosphoric acid (H_3_PO_4_) were procured from Merck, Mumbai, India. Chitosan, methyl-thiotetrazole (MTT), trypsin, dexamethasone, sodium beta-glycerol phosphate, and SEM grade gulteraldehyde were purchased from Sigma-Aldrich Chemical, St Louis, Mo, USA. Collagenase type I, 0.25% trypsin-EDTA, and Dulbecco's modified Eagle's medium (DMEM) were procured from Invitrogen, USA. Fetal calf serum and alpha-MEM were supplied by Hyclone and Pan-Biotech, respectively.

### 2.2. Preparation of Nano-Hydroxyapatite (n-HAp)

A concentration of 0.5 M stock solution each of Ca(NO_3_)_2_ · 4H_2_O and (NH_4_)_2_HPO_4_ was prepared in distilled water. Both solutions were taken in such amounts that Ca : P molar ratio was maintained at 1.67. Triethanolamine (TEA) was used in conjunction with Ca(NO_3_)_2_ · 4H_2_O solution (Ca^2+^ : TEA  =  1:0.5) as a capping agent to control the particle growth during precipitation. The pH of both calcium nitrate and DAHP solutions was maintained at ~11-12. (NH_4_)_2_HPO_4_ was added drop-wise to the mixture of Ca(NO_3_)_2_ · 4H_2_O and TEA and vigorously stirred at room temperature using a mechanical stirrer (2500 rpm). The pH of the reacting mixture was also maintained in the range of 11-12 by adding NH_4_OH solution gradually. The process was continued up to 6 hours. The gelatinous precipitate thus obtained was filtered by a centrifugal filtration process. The precipitate was washed with water thoroughly as well as with NH_4_Cl solution to remove the excess residual TEA, and dried at 90°C for 15 hours.

### 2.3. Synthesis of Chitosan Phosphate (CSP)

A mixture of approximately 2 g chitosan powder, 100 mL of 2% acetic acid solution, 6 g of orthophosphoric acid was taken in a three naked-round bottomed flask equipped with a stirrer, condenser, thermometer, and nitrogen gas inlet tube. The mixture was heated up to 80°C under constant stirring until dissolution of chitosan powder and then heated to reflux. After 2 hours, the resultant solution was cooled and precipitated in excess methanol. Again, the precipitated gel was dissolved in water and all unreacted phosphate (H_3_PO_4_) and acetic acid were removed by repeated reprecipitation in excess methanol. Finally, the gel was collected and dried under vacuum oven at 80°C over night.

### 2.4. Synthesis of HAp/CSP Nanocomposite

At first, the CSP was dissolved in hot water, and then the nanoparticles were added slowly with vigorous stirring with the help of a mechanical stirrer at 3000 rpm. After addition of entire nanoparticles to the polymer solution, the resulting solution mixture was kept in a vacuum desiccator to remove the bubbles. Then, the mixture was heated in a water bath to evaporate water. The resulting slurry was poured into a glass petri dish and dried to make a film, keeping it in a vacuum oven at 90°C over night. After that, the film was cut into dumbbell-shaped samples. Six sets of HAp/CSP nanocomposites were prepared by adding 10 to 60 wt.% of n-HAp particles as filler.

### 2.5. Characterization

#### 2.5.1. Physicochemical Characterization

**(1) XRD Study**

The phase analysis of the HAp powder and composite samples was done by XRD (Model PW 1729, Philips, Holland) using 35 milliamps, and 40 kV current, with a monochromatic CuK_*α*_ (target) radiation (*λ* = 1.5405  Å) with a step size of 0.04°2*θ*, a scan rate of 0.02°2*θ*/s, and a scan range from 2*θ* = 10 to 60°.

**(2) FTIR Study**

The identification of functional groups in the HAp powder, matrix, and composite samples as well as the interfacial modification (i.e., nature of bonding between particles and the polymeric matrix) were analyzed by FTIR analysis (Model NEXUS870, FTIR, Thermo Nicolet, USA) within the scanning range 4000–400 cm^−1^.

**(3) TEM Study**

The morphology, particle size of HAp powder, and dispersion of particles in the polymer matrix were observed through a Phillips CM 200 transmission electron microscope (TEM) with an acceleration voltage 200 kV.

**(4) SEM Study**

The surface roughness of the nanocomposite samples was investigated by scanning electron microscopy (SEM) (Model JSM-5800, JEOL, scanning electron microscope, Japan).

**(5) Mechanical Testing**

The tensile testing of the composite films was carried out on a universal mechanical testing machine (Model H10KS, HOUNSFIELD, UK) at a crosshead rate of 50 mm/min.

#### 2.5.2. Biocompatibility Study

**(1) Cell Viability Study**

In vitro cytotoxicity study of the material was carried out by culturing murine fibroblast L929 cells in a contact mode. Briefly, L929 cells were cultured in Dulbecco's modified Eagle's medium (DMEM) and seeded on the sheets/films at their exponential phase of growth at a density of 10^5^ cells/cm^2^. The cells were allowed to attach to the films surface for 3 hours in 5% CO_2_ incubator at 37°C. Fresh DMEM medium supplemented with 10% fetal calf serum (FCS) was added to each well to keep the cell containing films submerged. The plates were incubated for 24 hours at 37°C in a humidified atmosphere of 5% CO_2_ in air. After 24-hour incubation, MTT (4 mg/mL) was added to each well at a strength of 10% (v/v) and incubated for further 4 hours at 37°C. Subsequently, the media containing MTT was removed, and 200 *μ*L of DMSO was added to dissolve the formazan crystals. The absorbance was measured using an ELISA plate reader (Biorad, USA) at 595 nm. Student paired *t*-test was performed to test for statistical significance, and a *P*-value of <.05 was determined to represent a significant difference.

**(2) Primary Murine Osteoblast Culture**

Primary murine osteoblast cells were obtained by serial enzymatic digestion of neonatal mice calvariae. Briefly, calvariae were dissected from 3-4 days old neonatal mice. After removing adherent mesenchymal tissue and periosteum, calvariae were subjected to five sequential 15-minute digestions in an enzyme mixture containing 0.05% trypsin and 0.1% collagenase type I at 37°C on a rocking platform. The first two fractions were discarded, and fractions 3–5 were collected and immediately chilled by the addition of cold DMEM containing 10% FCS. Released cells were pooled, centrifuged, resuspended in medium, and filtered through a 70-micron mesh. Cells were plated at 10^4^ viable cells/cm^2^ in six-well culture plates in DMEM containing 20% FCS. Then, 24 hours later, the medium was changed to Dulbecco's modified Eagle's medium with 10% FCS (basal medium), and cells were fed again after 3 days. The cells from third to fifth passages were used for the differentiation studies.

**(3) In Vitro Osteoblast Differentiation**

Confluent monolayers of osteoblastic cells were enzymatically lifted from the flasks using 0.25% trypsin in 4 mM EDTA. The cells were concentrated by centrifugation at 300 g for 10 minutes and resuspended in a known amount of media [22]. Cells were counted using hemocytometer and diluted to the desired concentration of cells in complete media containing alpha-MEM supplement with 10% FBS, 1% antibiotic-antimycotic solution, 10 *μ*M sodium beta-glycerol phosphate, 50 *μ*g/mL L-ascorbic acid, and 100 nM dexamethasone. Aliquots of 20 *μ*L of cell suspension were seeded onto the top of the films prewetted and equilibrated with complete media placed in the wells of 24-well plates resulting in a seeding density of 10^4^ cells/cm^2^. The films were left undisturbed in an incubator for 3 hours at 37°C to allow for cell attachment to the films, after which an additional 1 mL of complete media was added to each well. Medium was changed every 3 days. Cultures were maintained in a humidified atmosphere consisting of 5% CO_2_ at 37°C. Osteoblast proliferation was determined at 3, 7, 14 and 21 days. The films were gently washed using fresh medium to remove unattached cells and exhausted medium followed by MTT assay as described above.

**(4) Alkaline Phosphatase Assay**

Culture medium was removed from the films and the cells were washed twice with PBS. The cells were lysed with 250 *μ*L of Triton X-100 (0.01% in PBS) for 30 minutes at 4°C. The obtained homogenate was used for the measurement of alkaline phosphatase (AP) activity and total protein concentration. AP activity was determined by an assay based on the hydrolysis of p-nitrophenyl phosphate to p-nitrophenol. About 50 *μ*L of the Triton lysate was added to 125 *μ*L of active reagent containing 0.012 M p-nitrophenyl phosphate in 0.05 M diethanolamine, pH 9.8, and incubated for 30 minutes at 37°C. The reaction was stopped with 50 *μ*L of 2.5 M sodium hydroxide and the AP activity was determined by measuring the absorbance of p-nitrophenol at 405 nm using a Microplate Reader (Bio-Rad).

**(5) Osteoblasts Morphology and Their Nodule Formation**

Osteoblasts morphology and their nodule formation were investigated through scanning electron microscope (SEM) study. For SEM studies, the attached cells on the composite films were rinsed twice with PBS, and fixed with 250 mL of 2.5% glutaraldehyde in PBS for 30–60 minutes. After washing with PBS, dehydration was performed by slow water replacement using series of ethanol solutions (30%, 50%, 70%, and 90%) for 15 minutes with final dehydration in absolute ethanol for 30 minutes, allowing samples to dry at room temperature and under vacuum. The films were mounted on stubs and coated in vacuum with gold. Cells were examined with a JSM-5800, JEOL, scanning electron microscope, Japan.

## 3. Results and Discussion

### 3.1. Physicochemical Study

#### 3.1.1. XRD Study

**Figure 1:**
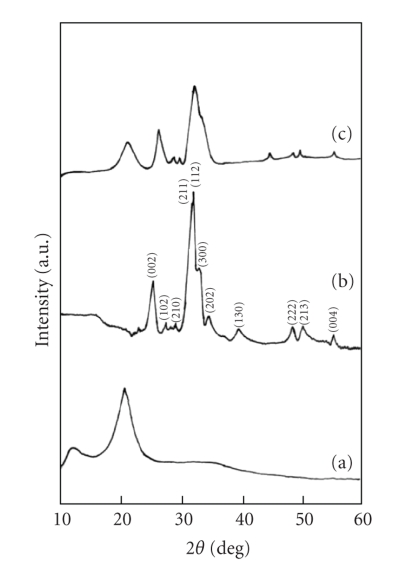
X-ray diffraction patters of (a) CSP, (b) HAp, and (c) HAp/CSP nanocomposite.

Figure 1(b) shows the X-ray diffraction pattern of the synthesized apatite powder. The d-values correspond to that of calcium hydroxyapatite (Ca_10_(PO_4_)_6_(OH)_2_) (JCPDS card no. 74-0566). Taking into account the broadening of each peak in XRD, mean crystallite size has been calculated using Scherrer's equation, that is, *D* = 0.9*λ*/*β*cos*θ*, where *D* is the average crystallite size in Å,  *β* is the peak broadening of the diffraction line measured at half of its maximum intensity in “radian,” *λ* is the wavelength of X-rays, and *θ* is the Bragg's diffraction angle. The mean crystallite size is found to be 15 nm. The approximate particle size of HAp powder is found to be 6–10 nm in diameter by 26–56 nm in length with needle-like acicular crystals as has been observed from TEM micrograph. The use of synthesized n-HAp powder with the CSP polymer as a matrix thus provides an effective means to produce nanocomposites.

Figure 1(c) shows the X-ray diffraction pattern of the HAp/CSP composite. The crystallite size of HAp in composite is found to be 12 nm. The crystallinity (*Xc*) of the pure HAp and HAp/CSP composite is determined by an empirical relation between *Xc* and *β*_002_ (i.e., $\beta_{002}\times \sqrt[{3}]{Xc}=K_{A}$) [23], where *Xc* is the crystallinity degree, *β*_002_ is full width of the peak at half intensity of (002) plane in degree −2*θ*, and *K_A_* is a constant (0.24). The *Xc* for pure HAp powder is 0.15, and in the HAp/CSP composite, it is found to be 0.11. The crystallinity of the n-HAp particles as well as CSP polymer has decreased after composite formation as shown in Figure 1 indicating that crystal structures of both HAp and CSP have changed after composite formation, which may be resulted from the interface binding between particles and matrix. The XRD peaks, (002) and (211) have shifted to higher 2*θ* values in case of HAp/CSP composite as compared to pure HAp (i.e., from 2*θ* = 25.98 to 26.57 and 31.89 to 33.46, resp.), which is possibly due to compression from the contracting polymeric matrix through interfacial bonding. Simultaneously, the crystalline peak of CSP polymer at 2*θ* = 20.8 has shifted to 21.6, whereas, another crystalline peak of CSP at around 2*θ* = 10.5 has disappeared in the composite (Figure 1(c)). The shift and decrease in crystallinity of each peak of the polymer as well as HAp after composite formation clearly indicate the presence of bonding between HAp particles and polymer matrix. The peak at 32.49 [300] of n-HAp is weakened after composite formation, which also indicates the participation of n-HAp in bonding with the polymer.

#### 3.1.2. FTIR Study

**Figure 2:**
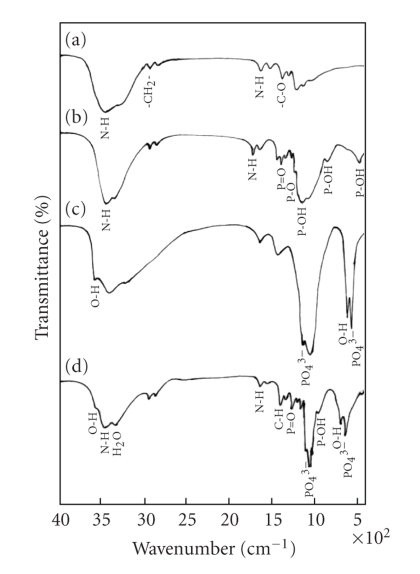
FTIR spectra of (a) chitosan, (b) chitosan phosphate, (c) HAp powder, and (d) HAp/CSP nanocomposite.

Figure 2(b) shows the FTIR spectrum of CSP having characteristic peaks at 2934 and 2850 cm^−1^ for asymmetric and symmetric stretching of methylene (–CH_2_–) groups, respectively. On the other hand, the bands at around 1544 and 1620 cm^−1^ are for N–H stretching in CSP. The bands at around 1088 and 1047 cm^−1^ are attributed to the C–O–P stretching and phosphorylated hydroxyl group, whereas, the characteristic peaks at around 991 and 493 cm^−1^ are attributed for P–OH groups in CSP polymer. The bands at around 1100 to 1250 cm^−1^ are due to P–O, P=O stretching of phosphate group. The bands at 632 and 3571 cm^−1^ are for structural OH groups in the n-HAp crystals (Figure 2(c)). The bands at 1092, 1030, and 604 cm^−1^ indicate the presence of PO_4_^3−^ group, and the spectrum also indicates the presence of H_2_O (3422 and 1641 cm^−1^) in HAp crystals. The peaks at 1047 and 493 cm^−1^ for P–OH groups in CSP have disappeared as shown in the Figure 2(d) for HAp/CSP composite, which may be due to the formation of polyphosphonate salt [14]. The peak intensity of P–OH at 991 and 1088 cm^−1^ in CSP polymer has decreased after composite formation, which evidences that the P–OH groups of CSP polymer have taken part in bonding with hydroxyapatite. Similar trend is seen in case of OH (632, 3571 cm^−1^) groups of HAp crystals, which is an indication of participation in bonding of HAp with polymer. Most of the peaks either in polymer or in HAp have shown clear shift after composite formation (Table 1). Thus, a comparison of the FTIR analysis has indicated that there is a chemical bonding at the nanoparticle-polymer interface.

#### 3.1.3. TEM Study

**Figure 3:**
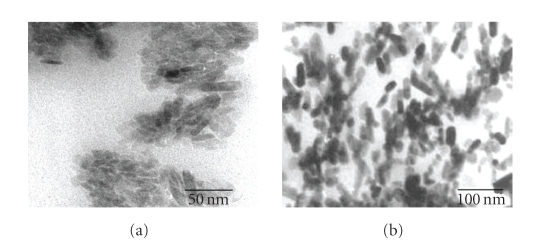
TEM micrographs of (a) HAp powders and (b) HAp/CSP nanocomposite (40% w/w).

Figure 3(a) shows the TEM micrograph of the synthesized n-HAp powder. The micrograph depicts the acicular needle-like crystals of HAp powder in nanometer range, having 6–10 nm in diameter by 26–56 nm in length. The micrograph shows the presence of agglomerations among the particles, which are due to high specific surface energy of n-HAp particles resulting in their aggregations. Nanosized HAp particles with homogeneous dispersion are well identified in case of HAp/CSP composite (Figure 3(b)). The particle size of HAp is also controlled by the polymer (CSP) in composite as depicted in the TEM micrograph.

#### 3.1.4. SEM Study

**Figure 4:**
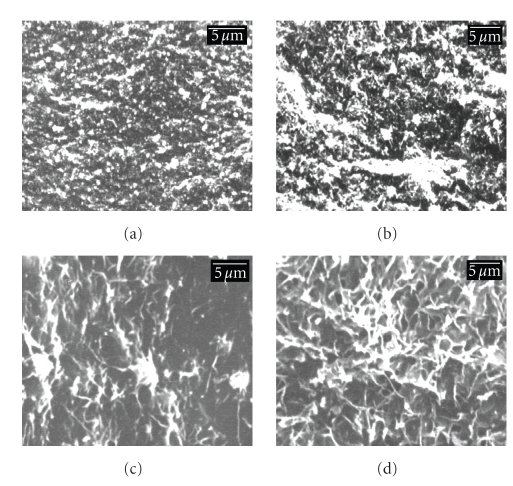
SEM micrographs of HAp/CSP nanocomposite samples with various contents of n-HAp particles (a) 20% (w/w), (b) 30% (w/w), (c) 40% (w/w), and (d) 50% (w/w).

Figure 4 shows the SEM micrographs of fracture surface of the composite samples with different contents of n-HAp particles. The micrographs depict that with increase in amount of HAp particles loading, the surface roughness for the composites is increased.

#### 3.1.5. Mechanical Properties Study

The tensile strength and modulus of the composites are found to be increased with increase in amount of HAp contents (Table 2). In 30% (w/w) n-HAp content, the increase in tensile strength and modulus has been found to be 100.4% and 190.8% than pure CSP polymer, respectively. In 40% (w/w) HAp loading, the tensile strength and modulus are increased by 140.8% and 225.5%, respectively. With 50% (w/w) HAp content, the tensile strength started to decrease, whereas, modulus is increased by 381.5% over pure CSP polymer. The results are summarized in Table 2. From the results, it is clear that the mechanical properties have been improved significantly with high nanoparticle loading capability, which means that the composite tends to be used as a biomaterial with high osteoconductivity [24]. The enhancement in tensile strength and modulus in case of HAp/CSP nanocomposite could be attributed to the excellent bonding at the nanoparticle-polymer matrix interface through the pendant phosphate groups of CSP.

### 3.2. Biocompatibility Study

#### 3.2.1. Cell Viability Study

**Figure 5:**
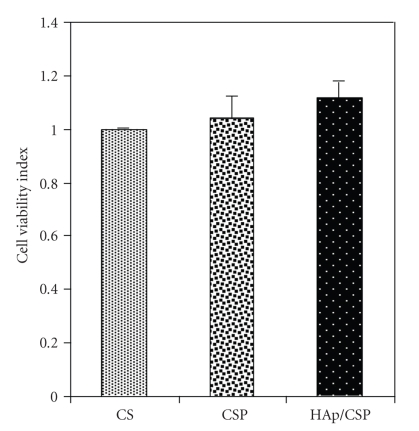
Cell viability test using murine fibroblast cell line L929 on CSP and HAp/CSP films, CS was taken as control (*n* = 3).

A cell line of murine fibroblast (L929) has been selected for the MTT assay test. The difference in cell viability index is shown in Figure 5 of the composite samples of different concentrations, as compared to the control tissue culture plate. The statistical analysis (Student's *t*-test) has indicated that the difference in cell viability index is insignificant. Hence, the developed composite is cytocompatible.

#### 3.2.2. In Vitro Osteoblast Differentiation Study

**Figure 6:**
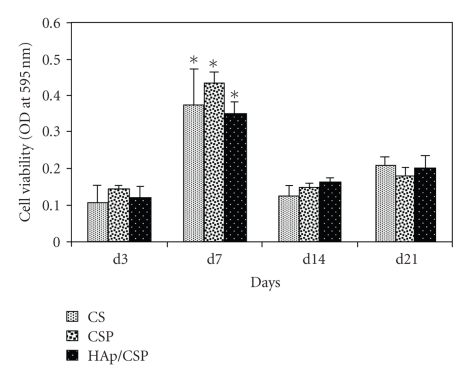
Osteoblast cell proliferation on CSP and HAp/CSP films, CS was taken as control. Significant increase in cell number (*P* < .001) was found at day 7 in all the variations followed by retardation of growth phase.

The neonatal mouse calvarial osteoblasts have been used to study the development of bone cells on CSP and HAp/CSP nanocomposite film surfaces. The results of cell proliferation of murine calvarial osteoblasts cultured on CSP and HAp/CSP nanocomposite films show a cell proliferation during the first 7 days (Figure 6). The proliferation phase follows a growth retardation after 7 days due to high confluence of cells on the film surfaces. The results show that HAp/CSP nanocomposite is an appropriate biomaterial for osteoblast proliferation.

#### 3.2.3. Alkaline Phosphatase Enzyme Activity Study

**Figure 7:**
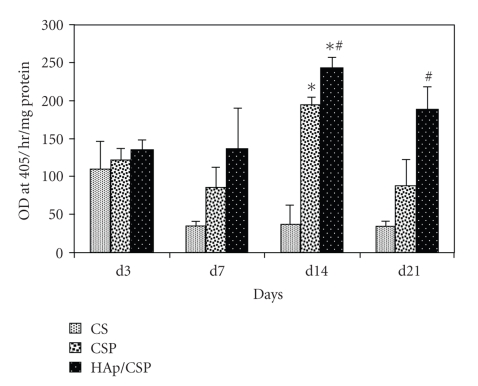
AP activity of osteoblast cells grown on CSP and HAp/CSP films, CS was taken as control. Both in case of CSP and HAp/CSP enzyme activity increased up to day 14 (*P* < .001). HAp/CSP nanocomposite is proven a better support for osteoblast differentiation than CSP alone (*P* < .01).

The differentiation process of the osteoblast cells on CSP and HAp/CSP nanocomposite has been analyzed by the alkaline phosphatase (AP) enzyme activity, which is an early osteogenic marker. The highest enzyme activity is achieved at day 14 in both materials (Figure 7). The AP activity shown by osteoblast cells growth on HAp/CSP nanocomposite is significantly higher than that on CSP surface.

#### 3.2.4. Osteoblasts Morphology and Their Nodule Formation

**Figure 8:**
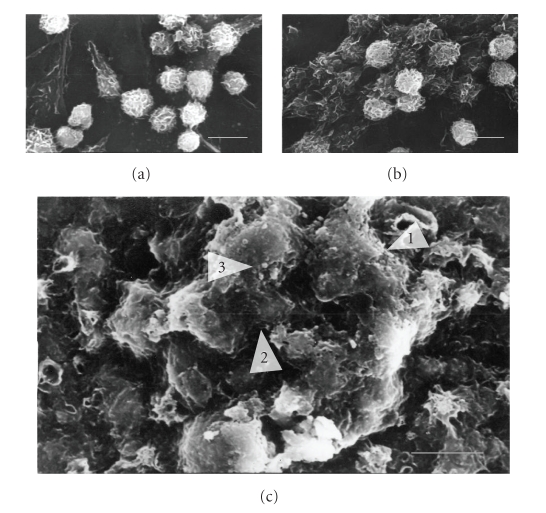
SEM images of osteoblasts grown on different matrices at day 14 on (a) chitosan (CS), (b) chitosan phosphate (CSP), (c) HAp/CSP nanocomposite. Scale bar of each image is 10 *μ*m. Arrow “1” shows a nodular cell aggregate, arrow “2” shows the extracellular matrix secreted by the cells in around themselves, and arrow “3” shows a visible mineral nodule.

Further, SEM examination has been conducted at day 14 to observe the bone formation process. Osteoblast cultured on the chitosan films reveals a round shape and stayed segregated. Secreted extracellular matrix or mineralization is not observed there (Figure 8(a)). Whereas, osteoblast cultured on chitosan phosphate films has shown tight aggregation and some mineralization (Figure 8(b)). However, osteoblast cultured on HAp/CSP nanocomposite shows a characteristic growing in multiple-layer patterns and tends to form a nodular cell aggregation (Figure 8(c)). Nodules contain densely packed cells are embedded within highly mineralized extracellular matrix secreted by them. SEM and AP enzyme activity studies indicate that incorporation of hydroxyapatite increases in vitro osteogenicity of chitosan phosphate films.

## 4. Summary and Conclusions

A novel bioanalogue HAp/CSP nanocomposite with uniform dispersion of n-HAp particles has been synthesized successfully following a solution-based chemical methodology with an appreciable improvement in mechanical properties and minimal surface defects. XRD and FTIR analyses clearly confirm the presence of interfacial bonding interaction between the filler and matrix. The use of CSP acts as a coupling/anchoring agent and provides a significant platform for better dispersion of nanoparticles in the polymer matrix through its pendant phosphate groups. Cytotoxicity test confirms that the developed composite is cytocompatible. Primary murine osteoblast cell culture study proves that the HAp/CSP nanocomposite is osteocompatible and highly osteogenic in vitro. The uniform dispersion of HAp particles using CSP through its pendant phosphate groups followed by improvement in mechanical properties of the composite is an important parameter in order to improve the bioactivity. Therefore, the developed HAp/CSP nanocomposite may be potentially used in bone tissue engineering applications.

## Figures and Tables

**Table 1:** FTIR peak positions of various functional groups before and after composite formation.

Functional groups	IR peak positions (cm^−1^)			
	CS	CSP	HAp particles	HAp/CSP
PO_4_^3−^	—	—	1092, 1030, 604	1079, 1025, 602
P–OH	—	1088, 1047, 991, 493	—	1070, disappeared, 984, disappeared
O–H bending	—	—	632, 3571	625, 3563
O–H stretching	3430	3427	3422	3402
N–H	3467, 1622, 1544	3465, 1620, 1544	—	3462, 1620, 1543

**Table 2:** Mechanical properties of HAp/CSP nanocomposite samples. Number after  ±  sign corresponds to the standard deviation.

n-HAp particles loaded (w/w)	Tensile strength (MPa)	Young's modulus (MPa)	Elongation at break (%)
0%	6.37 ± 0.55	74.45 ± 0.94	72.87 ± 2.66
10%	7.57 ± 0.29	147.87 ± 1.23	47.99 ± 2.17
20%	10.43 ± 0.87	184.89 ± 2.77	33.95 ± 0.66
30%	12.77 ± 1.06	216.56 ± 3.45	30.84 ± 1.84
40%	15.34 ± 0.47	242.38 ± 2.76	22.28 ± 2.58
50%	11.89 ± 0.78	358.54 ± 4.85	20.77 ± 1.42
60%	10.65 ± 0.49	302.68 ± 3.97	18.76 ± 0.95
